# Three-Dimensional Screen: A Comprehensive Approach to the Health Monitoring of Zebrafish

**DOI:** 10.1089/zeb.2015.1200

**Published:** 2016-07-01

**Authors:** Jean-Philippe Mocho

**Affiliations:** Mill Hill Laboratory, The Francis Crick Institute, London, United Kingdom.

## Abstract

Considering the numbers of zebrafish held in the laboratories, it is relevant to develop some tools to monitor the health of the animals, as well as their biotope. Environmental samples can be used to detect aquatic pathogens. Comprehensive health monitoring would thus seek pathogens in three dimensions of the animals and microbes' habitat: the fish, the sludge, and the water. This three-dimensional approach is called the 3D screen and it introduces some complementary tools to routine sentinel screening. For example, sludge and sump swabs analyses allow an efficient detection of pathogens at a low cost and with a fast turnover. These assays are particularly useful in cases of *Pseudocapillaria tomentosa* infestation or *Mycobacterium haemophilum* outbreak. Indeed, such a broader choice of diagnostic tests gives flexibility for the veterinarian to investigate *Mycobacterium* spp. presence in the water systems and fish colonies. Some other robust additional analysis, like the mortality rate monitoring, quickens the decision-making process. The 3D screen describes how this new toolbox can be used efficiently to monitor laboratory fish health.

## Introduction

Research benefits from the health monitoring of laboratory animals.^1,2^ Detecting the presence of pathogens leads to a better interpretation of experimental results. Monitoring the presence of pathogens allows actions to be taken to improve health and reduce mortality due to disease, as well as improving cost effectiveness of research. Knowing the health status helps to define quarantine procedures when exchanging colonies to refresh wild-type genetic pool or to import new genetically altered lines.

Zebrafish (*Danio rerio*) are no exception.^3–6^ The health screening often consists in setting sentinels prefiltration and/or postfiltration. Sentinels and colony animals are tested by histopathology for a general pathology screen and/or by polymerase chain reaction (PCR) to identify a specific pathogen or to screen for a defined panel of pathogens. Exposing prefiltration sentinels to contaminant may increase the animal carer's workload when the sentinels are set in an independent tank receiving sump water. Otherwise, adding a sentinel tank to the recirculating system is sometimes an expensive engineering task. Sentinels need time to be exposed to present pathogens and that restrict the flexibility to test the colonies and the systems when a problem appears. It is not practical when screening imported fish in quarantine. Sending colony animals and sentinels in numbers to detect less prevalent pathogens increases the number of animals used.

We propose in this study a more comprehensive and flexible approach to health monitoring of zebrafish by detecting pathogens in three dimensions of their habitat: the fish, the sludge, and the water. Fish health is monitored by monthly report of mortality. Colony animals with or without clinical signs (found dead or euthanized) are screened to explore the mortality data. Prefiltration sentinels are exposed to contaminant from sumps and screened. Tank sludge is screened for the presence of pathogens, for example, *Pseudocapillaria tomentosa* eggs.^7^ The water and biofilm are sampled for bacteriology test, for example, *Mycobacterium* spp. speciation.^8^

This 3D screen proposes a three-dimensional approach to fish health monitoring and it gives some flexibility. When an increase in mortality is detected and sentinels are being exposed, testing the water and the sludge is still a valuable option and it proposes a more comprehensive set of results than just testing colony fish on the system randomly. Water, biofilm, and sludge samples are not time consuming samples to take. Such comprehensive approach may reduce the numbers of animals used to detect the presence of some pathogens. For example, *Mycobacterium* spp. can be identified, thanks to the biofilm. Analysis of the sludge is a useful tool in quarantine to screen for imported animals without sacrificing any.

The 3D approach is used at the Francis Crick Institute, London, United Kingdom, where the zebrafish facilities mainly support developmental biology projects. The Institute was opened in April 2015 and has been operating across multisite with zebrafish held in two locations from legacy National Institute of Medical Research (MH) and London Research Institute (LIF). Due to the historical development of each site independently over the last 30 years, there is a wide diversity of practices and systems in use. Therefore, we will only detail the relevant information to describe the 3D screen and how we used it to investigate outbreaks in two systems, namely room 8 system 2 at MH and Aquarium A at LIF. We will see how this comprehensive approach to diagnostics helped in cases of *Mycobacterium haemophilum* outbreak and *P. tomentosa* infestation. In addition, we will describe the advantages of looking for *Mycobacterium* spp. in the environment rather than in the fish. Finally, we will detail how the 3D screen can help the decision-making process.

## Materials and Methods

### Overview of the fish facilities

Across the two institute sites, the fish are housed in 25 independent recirculating aquaculture systems (RAS) all fitted with mechanical filters, UV, biofilters, and sometimes with a carbon filter. Altogether, there are 10 different models of holding systems to keep 25,000 adult zebrafish. Most systems, like system 2 in room 8 at MH, are supplied with reverse osmosis water (RO) and aim for a conductivity of 500 μs, pH 6.5–8.0, temperature 26–28°C, Total Ammonia Nitrogen below 0.6 ppm, Nitrite below 0.5 ppm, and Nitrate below 100 ppm (Table 1). System 2 was made by Aqua Schwarz GmbH, Gottingen, Germany. Aquarium A at LIF is homemade and it is supplied with filtered and dechlorinated London city water. Aquarium A water parameters are 1300 μS conductivity and pH 8.4.

**Table 1.** Example of Water Quality Parameters for System 2 and Aquarium A

			*mg/L*					
*System*	*pH*	*Conductivity (μS)*	*TAN*	*Nitrite*	*Nitrate*	*GH*	*KH*	*Water supply*
System 2	7.0	500	0	0	73	51	36	RO
Aquarium A	8.4	1300	0	0	120	306	196	MAINS

GH, hardness; KH, alkalinity; RO, reverse osmosis; TAN, total ammonia nitrogen.

### Key husbandry elements

The zebrafish are only used for egg production, and animals with any clinical sign or over 24 months of age are euthanized. A database to detect lines with adverse effects is in development.

The fish are kept on a constant 14 h on light cycle/10 h off light cycle. Wild-type fish are bred by mass spawning to supply large clutch to researchers. Embryos are kept in incubators at 27–28°C until day 5 postfertilization. At that stage, 50 larval are set on the RAS in a 3.5 L tank and they are fed paramecium and ZM-000 (Zebrafish Management Ltd, Winchester, United Kingdom) twice daily. Brine shrimp is fed once daily from day 12. Paramecium is stopped at day 25. The ZM pellet size is increased to ZM-100 at day 19, ZM-200 at day 33, and ZM-300 at day 48. At 8 weeks of age, the fish are split in groups of 15 per 3.5 L tank and they are fed SAFE caviar 300–500 (SAFE, Augy, France) twice daily following a diet transition period with a reduction of brine shrimp supply to once per week.

Regarding biosecurity, the nets are soaked following each use in a solution containing 5 mL of 0.5% NaOCl solution per liter for 15 min. Then, they are thoroughly rinsed and heat treated at 90°C for 60 min. All imported fish are reared in quarantine.^2^ Only the eggs are introduced to our conventional systems following surface sanitization according to the ZIRC bleaching protocol.^9^

### The 3D screen

The 3D screen looks for pathogens in three dimensions of the fish and microbe habitat as follows: the fish, the sludge, and the water.

#### Detecting pathogens in zebrafish

To define mortality rates, animal carers monitor the health of the animals on a daily basis, they euthanize any sick fish, and they record any death of zebrafish older than 8 weeks of age. For each RAS, the number of fish dead in a month is divided by an estimation of the number of fish held on the RAS. A census is performed several times a year and the population size estimation is adjusted according to transfers and mortality.

To screen each room, prefiltration sentinels are used routinely. Due to space and biosecurity constraints, the quarantine system has its own tank of sentinel on the RAS and sump water is added daily to this tank.

Otherwise, sentinels are kept in stand-alone tanks. The density is reduced to 1–2 fish per liter—typical 10–12 fish in an 8 L tank. The fish are fed once a day and are exposed to all adult diets. Biomedia from the screened systems are added to the sentinel tank to expose the sentinels to this flora and to control better the water quality. Sentinel tank water is replaced on Monday, Wednesday, and Friday by sump water from the screened systems. To optimize *Mycobacterium* spp. detection,^4,10^ the sentinels are exposed for 4 months and the sentinel screen is performed two to three times a year.

The sentinel group is selected to replicate the aquarium dominant genetic background and to increase the sensitivity for pathogens that are more prevalent in one gender and/or age category.^11^ The sentinels are wild type (mainly AB): at least one female and one male below 6 months of age, one female and one male between 6 and 12 months, and one female and one male above 18 months of age.

When sampling is due, the sentinels are euthanized by immersion in an overdose solution of 2-phenoxyethanol (3 mL/L) for 10 min. For some fish, wet mounts^12^ (gill, mucus, scale, and fin) and in-house histopathology are performed (at least hematoxylin and eosin and acid-fast staining). These techniques help for the surveillance of unexpected and/or unknown pathogens. A minimum of three sentinels are sent to a commercial diagnostic laboratory for some specific PCR assays.

Zebrafish are often pooled for PCR and the list of tested pathogens is sometimes as comprehensive as possible: *M. haemophilum*, *Mycobacterium marinum*, *P. tomentosa*, *Edwardsiella ictaluri*,^13^ *Ichthyophthirius multifiliis*,^14^ *Piscinoodinium pillulare*,^15^ *Pleistophora hyphessobryconis*,^16^ *Flavobacterium columnare*,^17^ infectious spleen and kidney necrosis virus,^18^ *Mycobacterium abscessus*, *Mycobacterium chelonae*, *Mycobacterium fortuitum*, *Mycobacterium peregrinum*, and *Pseudoloma neurophilia.*

#### Detecting pathogens in the sludge

**FIG. 1.**
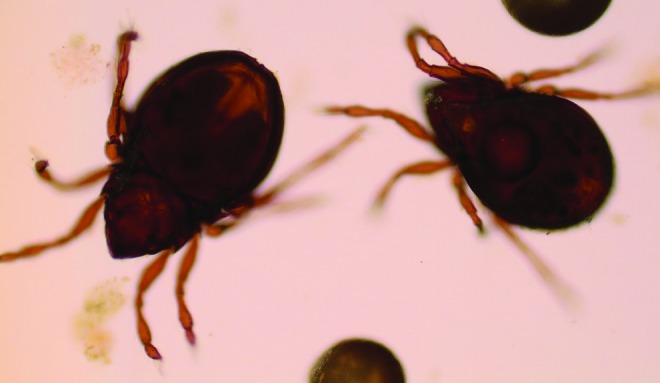
Arthropods detected by microscopy during sludge analysis (40×).

**FIG. 2.**
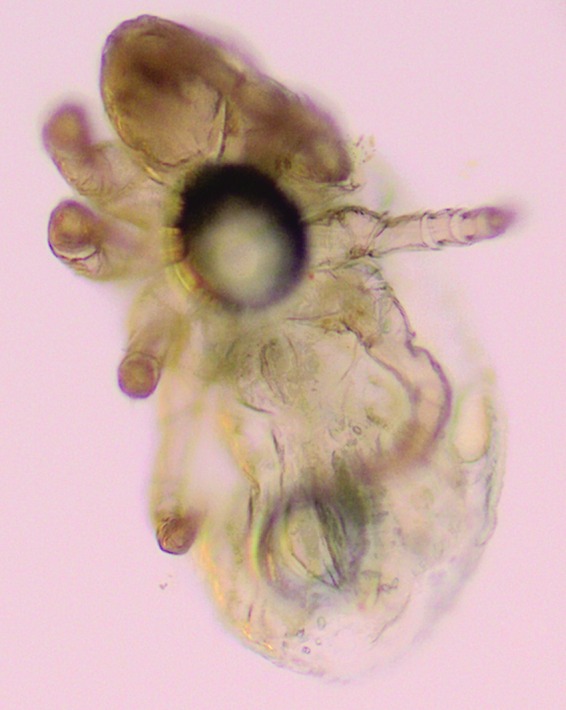
Arthropods detected by microscopy during sludge analysis (40×).

**FIG. 3.**
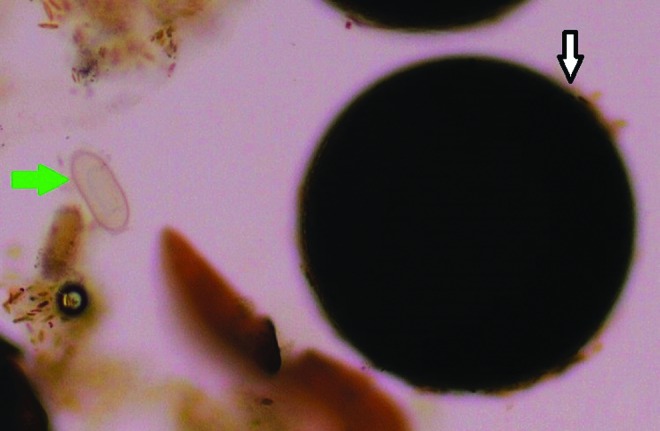
*Pseudocapillaria tomentosa* egg next to artemia egg as seen during microscopic screening of sludge (200×). *Green arrow* = *P. tomentosa* egg; *white arrow* = artemia egg.

**FIG. 4.**
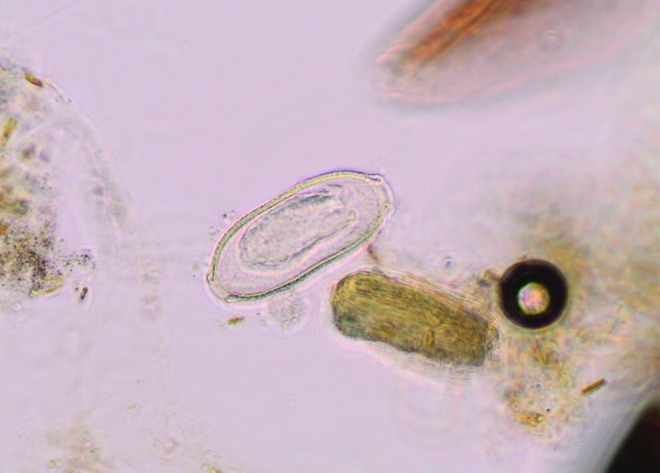
Zoom on *P. tomentosa* egg from Figure 3 (400×).

The analysis of the sludge can be performed to monitor the diverse population in the RAS biotope: algae, arthropods (Figs. 1 and 2), and mycobacterial biofilm. In this study, we detail the technique to detect *P. tomentosa* eggs^7^ (Figs. 3 and 4).

A 60 mL syringe is used to aspirate the sludge at the bottom of a holding tank. The sample is divided in 15 mL tubes and centrifuged at 1500 rpm for 10 min.^19^ The tubes are decanted and filled in half with sugar saturated solution (454 g granulated sugar mixed in 355 mL hot water). The sediment is stirred well before placing the tubes in the centrifuge swinging buckets. More sugar saturated solution is added to reach the top of the tube. A cover glass is set on top of the tube and in contact with the sugar saturated solution. The tubes are centrifuged at 1500 rpm for 10 min. To read the assay, the cover glass is lifted and placed on a glass slide.

A microscope is used to seek *P. tomentosa* eggs. They are easily identifiable with their bipolar plugs (Fig. 4) and their size (57–78 μm long and 27–39 μm diameter).^20,21^

#### Detecting pathogens in the water

Water samples can be used to detect *P. neurophilia* as described by Whipps and Kent.^22^ In this study, we describe how to monitor the RAS bacterial load and to specify the mycobacterial biofilm.

To monitor husbandry and UV filtration, bacterial counts of system water are performed thrice a year. The samples are taken aseptically in the sump or post-UV filtration. Bacterial counts are performed by serial dilutions on nutrient agar following 48 h of incubation at 30°C. Any suspicious growth during culture is identified and archived.

To identify the *Mycobacterium* spp. present in the systems, the sump wall is swabbed at the water surface on 5–10 cm.^8^ Thrice a year, commercial diagnostic laboratories test the swabs by PCR for presence of *Mycobacterium* spp. and, in case of positive, for identification of the six reported zebrafish pathogens^23^: *M. marinum*, *M. haemophilum*, *M. abscessus*, *M. chelonae*, *M. fortuitum*, and *M. peregrinum*.

## Results

The results of this health monitoring approach allowed us to define and confirm the health status of our two main zebrafish facilities. The following pathogens have never been detected: *M. marinum*, *E. ictaluri*, *I. multifiliis*, *P. pillulare*, *P. hyphessobryconis, F. columnare*, and infectious spleen and kidney necrosis virus. *P. neurophilia* is present on both sites. One site is infested by *P. tomentosa* and the results of Aquarium A are detailed below. The other site is contaminated by *M. haemophilum* and we will describe how the mortality recording results were used to manage an outbreak in room 8 system 2. First, we will compare the results on mycobacterial speciation by testing fish versus testing the sump swabs.

In 2015, for the routine screens of the main site, we sampled 83 fish and 14 sump swabs to be tested by PCR for *Mycobacterium* spp. identification by commercial diagnostic laboratories (Table 2). Only nine fish were PCR positive for *Mycobacterium* spp. and that includes three sentinel fish during a *M. haemophilum* outbreak. Note that other fish results specific to this outbreak are not included in this data. Out of the 14 sump swabs tested by PCR, 23 *Mycobacterium* spp. were identified. *M. fortuitum* was never identified in a fish, but it is the most prevalent finding in the sump. *M. haemophilum* was identified twice in quarantine and once in system 2.

**Table 2.** PCR Identification of *Mycobacterium* spp. in Fish and in Sump Swabs in 2015 at MH

*83 fish tested*			*14 sump swabs*	
*Positive*	*%*	*Mycobacterium* spp.	*%*	*Positive*
6	7	*M. chelonae*	57	8
3	4	*M. haemophilum*	29	4
0	0	*M. fortuitum*	71	10
0	0	*M. peregrinum*	7	1
0	0	*M. abscessus*	0	0
0	0	*M. marinum*	0	0

Percentage is obtained by dividing the number of positive results for each species by the number of tested samples. Note that this does not include the samples taken to confirm *M. haemophilum* infection in fish during the system 2 outbreak.

PCR, polymerase chain reaction.

While trends are reviewed continuously, our threshold to trigger veterinary investigation is a mortality rate above 1% for two consecutive months. In room 8, system 2 had an increased mortality rate of 1.81% in August 2015, thrice more than any other system in room 8 during that period (Table 3). Eight fish were euthanized and histopathology revealed widespread granulomas and acid-fast bacilli in four animals (50%). The sentinels had been euthanized in July and they had not displayed any clinical signs, although 50% (3/6) of them were PCR positive for *M. haemophilum*. In August, the room sumps and some fish from system 2 were sampled randomly. Fifty percent (8/16) of system 2 fish were PCR positive for *M. haemophilum*. System 2 sump was the only positive for *M. haemophilum* out of the five in the room. This pathogen had been identified in this facility previously,^24^ but it was the first time we had to take action against it and we shut the system down. Some new sentinels were set in August for the whole room. In the December screen, we could not detect *M. haemophilum* in the room sumps and in any sentinel (0/5). All along these investigations, the fish samples were PCR negative for *M. marinum*, *Edwardsiella ictaluri*, *F. columnare*, *P. hyphessobryconis,* and *P. neurophilia.* The sump samples were negative for *M. marinum.* And the general bacterial load of the systems did not display any significant increase, staying within the 10^3^–10^5^ cfu/mL range.

**Table 3.** Room 8 Mortality Records July and August 2015

*Room 8*	*Last census*	*July '15*		*August '15*	
*System*	*Total number of fish*	*Found dead+sick*	*Mortality (%)*	*Found dead+sick*	*Mortality (%)*
1	1778	9	0.51	5	0.28
2	2158	27	**1.04**	39	**1.81**
3	1653	4	0.24	7	0.42
4	1015	7	0.69	6	0.59
5	1945	10	0.51	7	0.36

For each system, monthly mortality percentage is obtained by dividing the number of found dead and euthanized zebrafish during the month by the estimation of the total number of fish in the system.

*Bold* figures signify mortality above 1.00%.

The first screen performed in Aquarium A on wild-type fish selected randomly revealed the presence of *P. tomentosa*. Two fish were positive by PCR and the results were supported by histopathology in these fish. More tests were requested to confirm the infestation. Altogether, 11 fish were tested by PCR in a commercial diagnostic laboratory and three were PCR positive. Concomitantly, 14 tanks due to be cleaned were selected for the presence of abundant sludge. Their sludge was analyzed and *P. tomentosa* eggs were seen by microscopy in 13 out of the 14 sludge samples (Table 4).

**Table 4.** Detection of *Pseudocapillaria tomentosa* in Aquarium A

*Fish tested by PCR*			*Tank sludge analyses*		
*Number*	*Positive*	*%*	*Number*	*Positive*	*%*
11	3	27	14	13	93

Percentage is obtained from number of positive results divided by number of tests.

## Discussion

The 3D screen approach allows us to health screen routinely our zebrafish colonies. The detection of *Mycobacterium* spp. seems to be helped with the sump swabs and this is a useful technique to define a health status. Regarding mycobacterial disease monitoring, the presence of *Mycobacterium* spp. in the environment is not significant. Only histopathology is able to determine if a fish PCR positive for *Mycobacterium* spp. is affected and this technique remains key to result interpretation. It is relevant to keep testing the fish to monitor the incidence of mycobacterial contamination since it can be linked with an increase of mortality, as in the outbreak described in this study.

This *M. haemophilum* example shows how a comprehensive approach can quicken the decision-making process. First, the mortality records supported the animal carers' suspicion that fish were not healthy in that system. However, we could not rely on the sentinels to confirm the suspicion. The high incidence of *M. haemophilum* in system 2 fish seemed unusual in our facilities and *M. haemophilum* is not often detected in the sumps either. It is the accumulation of data from the various sample types that sped the investigation up.

Similarly, the sludge analysis is a very fast tool to detect *P. tomentosa* eggs. The advantages of the technique compared with histopathology and PCR are the turnover (30 min) and the low cost. The high sensitivity reached in Aquarium A results reflects the bias at sample selection: the fish had been held in these tanks for a prolonged period. It is doubtful that such sensitivity would have been obtained with fish set in clean new tanks; this is a limit for the use of sludge analysis in quarantine.

Following investigation, it is likely that the increase of mortality and *M. haemophilum* incidence in room 8 system 2 was due to an attempt to feed the fish more. The animals were fed more than usual for a few months before the outbreak while the cleaning and husbandry routine remained unchanged. We suspect that this disturbed the biofilm and/or the fish flora and we changed our diet to the one described above. The general bacterial load monitoring did not allow any further confirmation of a husbandry issue or a general bacterial load increase. But, earlier in 2015, following this feed increase, we detected some cases of bacterial aero cystitis and septicemia. *Aeromonas* spp., *Pseudomonas* spp., and *Vibrio cholerae* non O1 were cultured from these fish.^25^ *M. haemophilum* would then strike later, like an opportunistic pathogen, as the infection often requires more time to develop.^4,10^

As shown in these examples, the flexibility of the 3D screen allows the use of lesser animals for screening. Depending on the aims of the screen, fish other than sentinels are sampled, for example, colony fish or escapees. Some more specific PCR panels are used to investigate clinical cases. According to the prevalence and pathogenicity of zebrafish pathogens, a higher number of fish are tested for the more relevant pathogens. For example, *P. tomentosa*, *M. marinum*, and *M. haemophilum* are tested in more animals than the other pathogens. But since we confirmed that *P. neurophilia* was present in all our stock, we have not spent any more money testing for it.

**FIG. 5.**
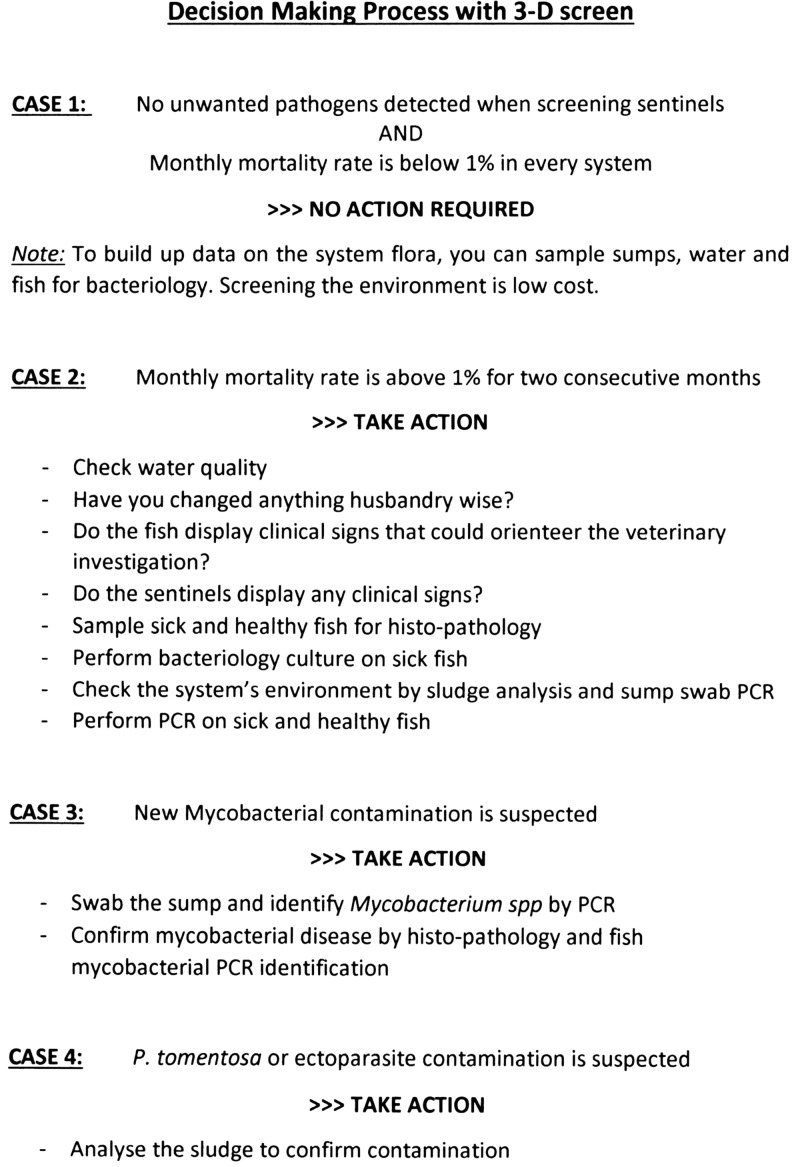
Decision-making process with 3D screen.

Figure 5 is a list of cases describing how the 3D screen approach supports the decision-making process. It shows how the flexibility of the system can be tailored to the facility. In absence of pathogens, the comprehensive approach can help reducing cost. In case of contamination or of increased mortality rates, the choice of assay is essential to confirm diagnosis and to analyze the impact on the system and colonies. This flowchart orients veterinary investigation toward the most adapted test (bearing in mind cost and turnover) for a few clinical cases.

## Conclusion

The 3D screen provides a robust process to adapt diagnostic methods to a particular situation. Not only screening one biotope or another, but also to tailor the PCR panel and to reduce the number of fish to sample. This flexibility is facilitated by the low cost of the sludge and water testing. In case of an outbreak, the 3D screen is a valuable tool to make a decision quickly. For the researcher, this approach of zebrafish health monitoring is an efficient means to define their colony's health status. This is becoming a requirement to share animals with collaborators^26,27^ and when presenting experimental data.
